# Commentary on: Preoperative MRI to Improve Aesthetic Outcomes in Secondary Mastopexy Augmentation: A Step-by-Step Approach

**DOI:** 10.1093/asjof/ojac075

**Published:** 2022-11-23

**Authors:** James D Namnoum

**Affiliations:** Private practice, Atlanta, GA, USA

See the Original Article here.

This paper attempts to illustrate the value of preoperative magnetic resonance imaging (MRI) in helping surgeons plan secondary augmentation/mastopexy.^1^ The authors contend that foreknowledge of the blood supply to the nipple-areolar complex especially in a setting of re-operative surgery in which the previous operative notes are unavailable will enable more aggressive evaluation of the nipple and consequent skin redraping skin and/or undermining. They acknowledge that most surgeons rely on clinical judgment when performing revisionary cases of this type and that complications may result from an assumption that the dominant circulation emanates from medial intercostal vessels arising from the internal mammary artery. They site 8 consecutive cases, the majority of which were reconstructive to demonstrate their point that preoperative MRI prior to revision augmentation/mastopexy prevents nipple-areolar compromise and wound healing complications (Video). Four cases were presented in detail differing in clinical detail. History, MRI data, operative plan, and 2-week results were shown. Patients 1 and 3 had prior augmentation and periareolar mastopexies, Patient 3 had multiple revision augmentations and a prior circumvertical mastopexy, and Patient 4 had nipple-sparing mastectomies and device reconstruction without any prior mastopexy. None of the cases required significant elevation of the nipple or extensive undermining of skin flaps. In most of the cases, there appeared to be significant blood supply from medial perforators and in Case 3 from medial and lateral perforators. I could see no reason why either perforating system would require disruption in any of these cases, so I remain unconvinced that the preoperative MRI was of clinical relevance here. I appreciate that some surgeons may take comfort from knowledge of the existing blood supply in revisional breast cases like the ones demonstrated but see no reason for the expense unless the surgeon intends to incise a nipple pedicle for rotation or transfer to the upper pole of the breast. In my experience, this is generally not warranted and unwise. There may be cases where a device was placed via an upper hemispheric periareolar incision dividing the superior blood supply with a periareolar mastopexy and the patient requires an exchange with nipple transposition and a more formal vertical or inverted T mastopexy. Preoperative MRI would clearly be beneficial in cases such as these.

## Supplementary Material

ojac075_Supplementary_Data
